# Supplementary figures and images for: Likelihood-Based Inference of B Cell Clonal Families
Source: PLoS Comput Biol. 2016 Oct 17;12(10):e1005086. doi: 10.1371/journal.pcbi.1005086 (PMC5066976; doi:10.1371/journal.pcbi.1005086)

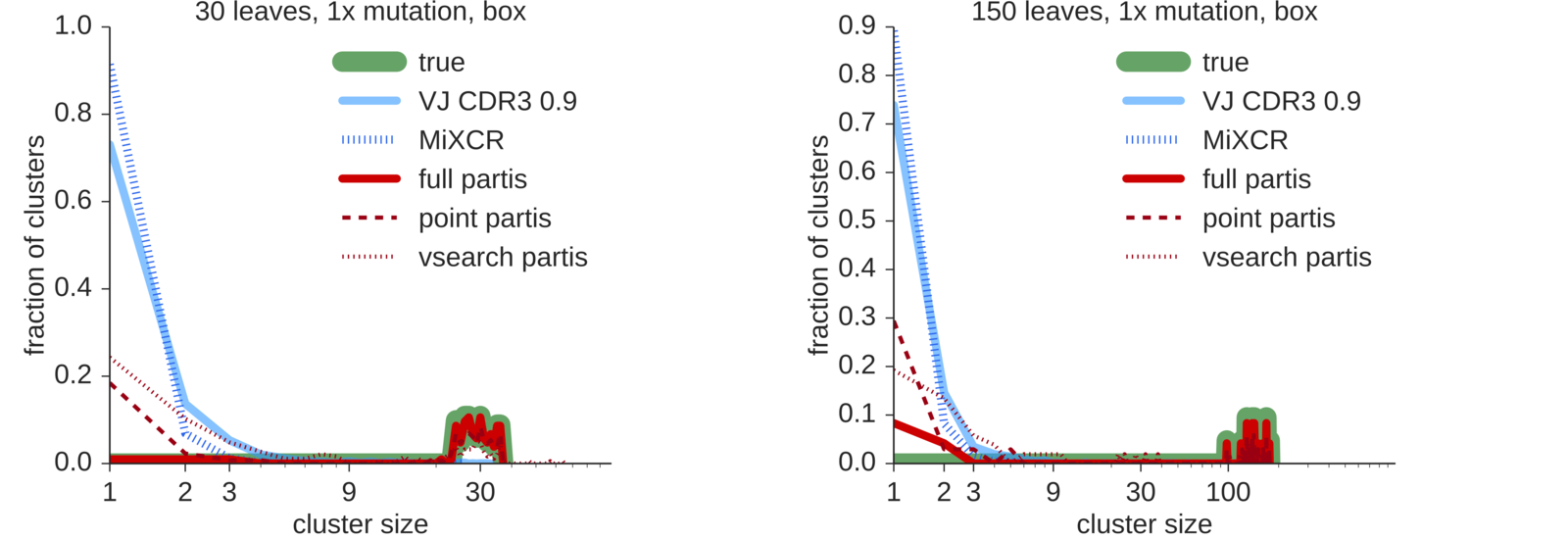

Supplement: S1 Fig — Results are shown for box distributions with mean 30 (left) and 150 (right). (TIFF) [file pcbi.1005086.s001.tiff]

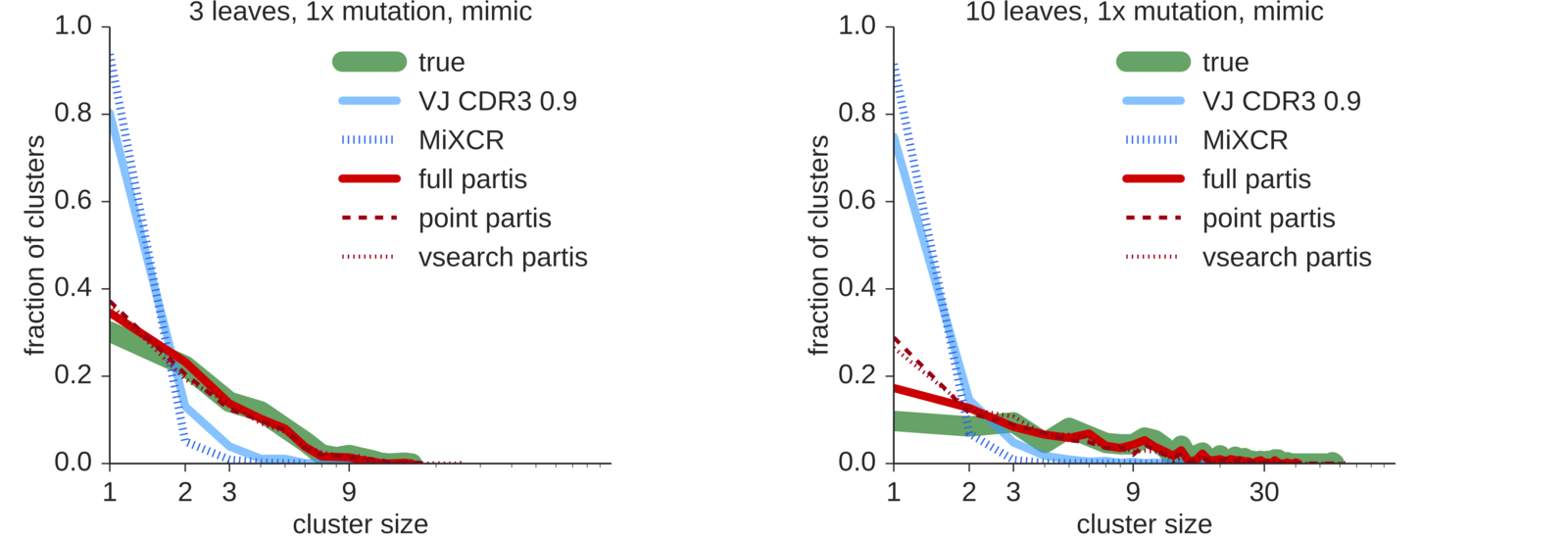

Supplement: S2 Fig — Results are shown for samples with reads of length 130 bases centered on the CDR3 (which mimics the Adaptive data set), for geometric cluster size distributions with mean 3 (left) and 10 (right). (TIFF) [file pcbi.1005086.s002.tiff]

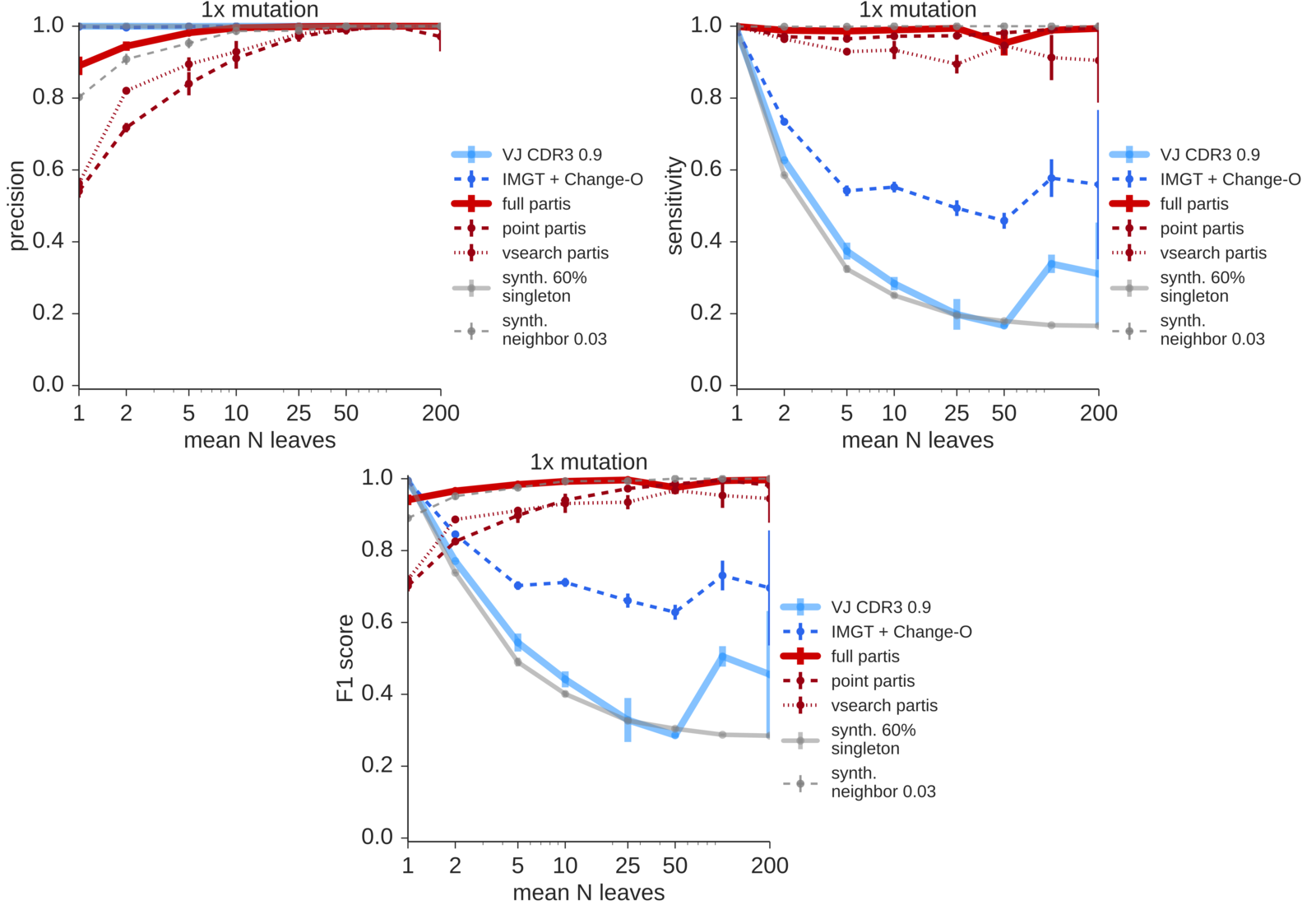

Supplement: S3 Fig — Results are on simulated sequences which span the entire V, D, and J segments; the number of leaves (BCR sequences per clonal family) is distributed geometrically with the indicated mean value. Precision measures the extent to which inferred clusters contain truly clonal sequences, while sensitivity measures the extent to which the entirety of each sequence’s clonal family appears in its inferred cluster. These plots also include synthetic partitions, which for purposes of comparison generate incorrect partitions starting from the true partition (“synth.”, see text for details). (TIFF) [file pcbi.1005086.s003.tiff]

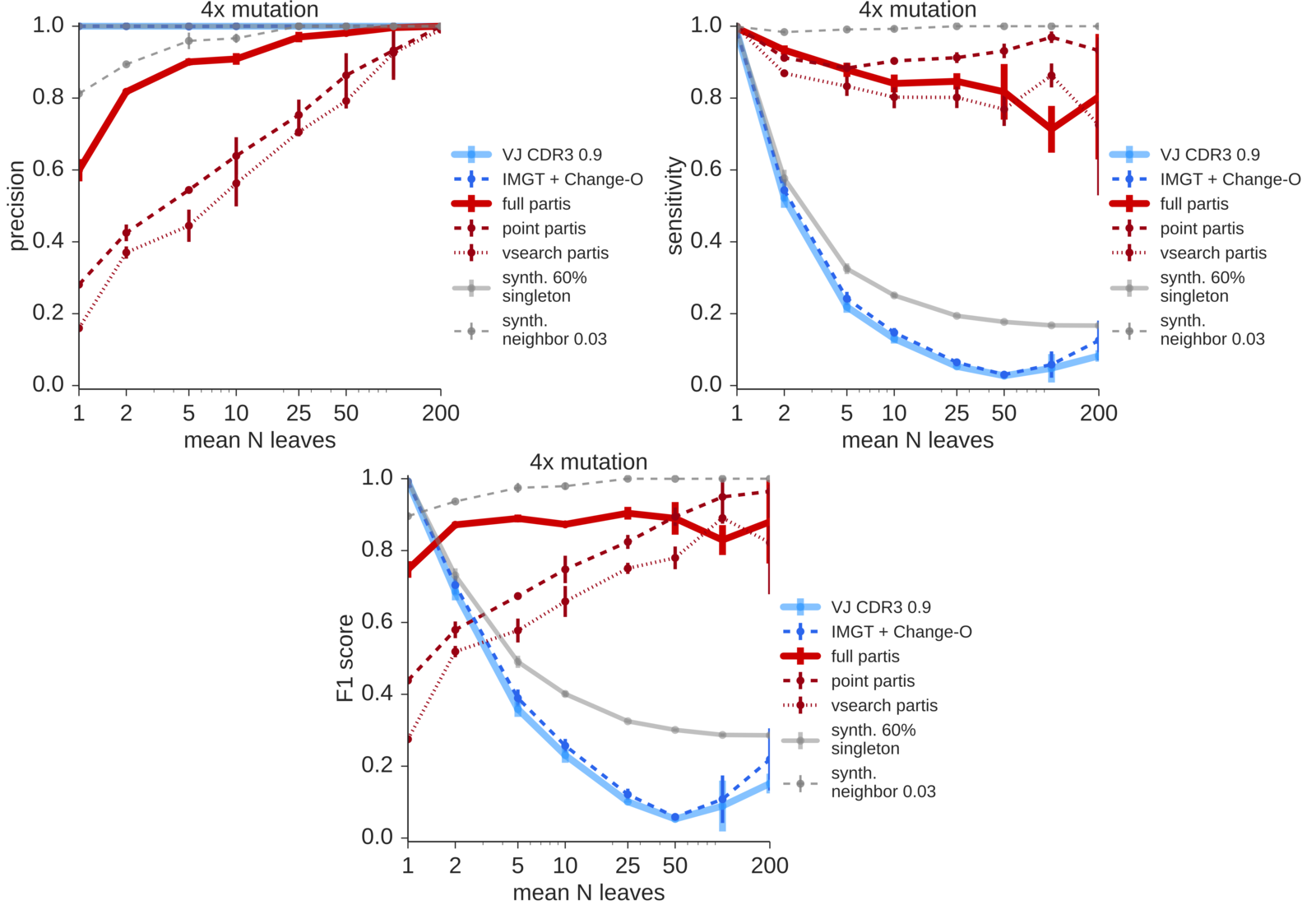

Supplement: S4 Fig — (TIFF) [file pcbi.1005086.s004.tiff]

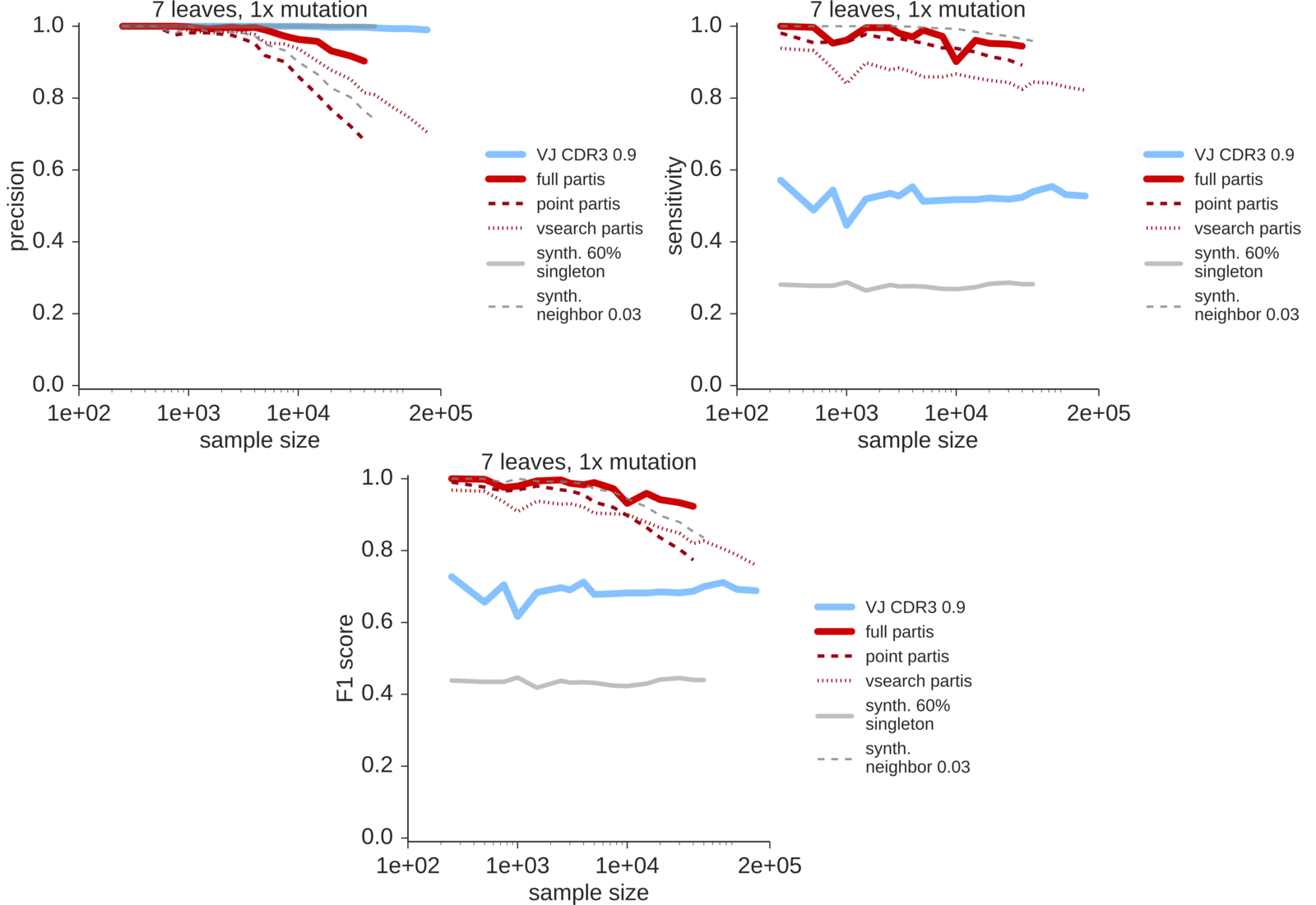

Supplement: S5 Fig — See S3 Fig. (TIFF) [file pcbi.1005086.s005.tiff]

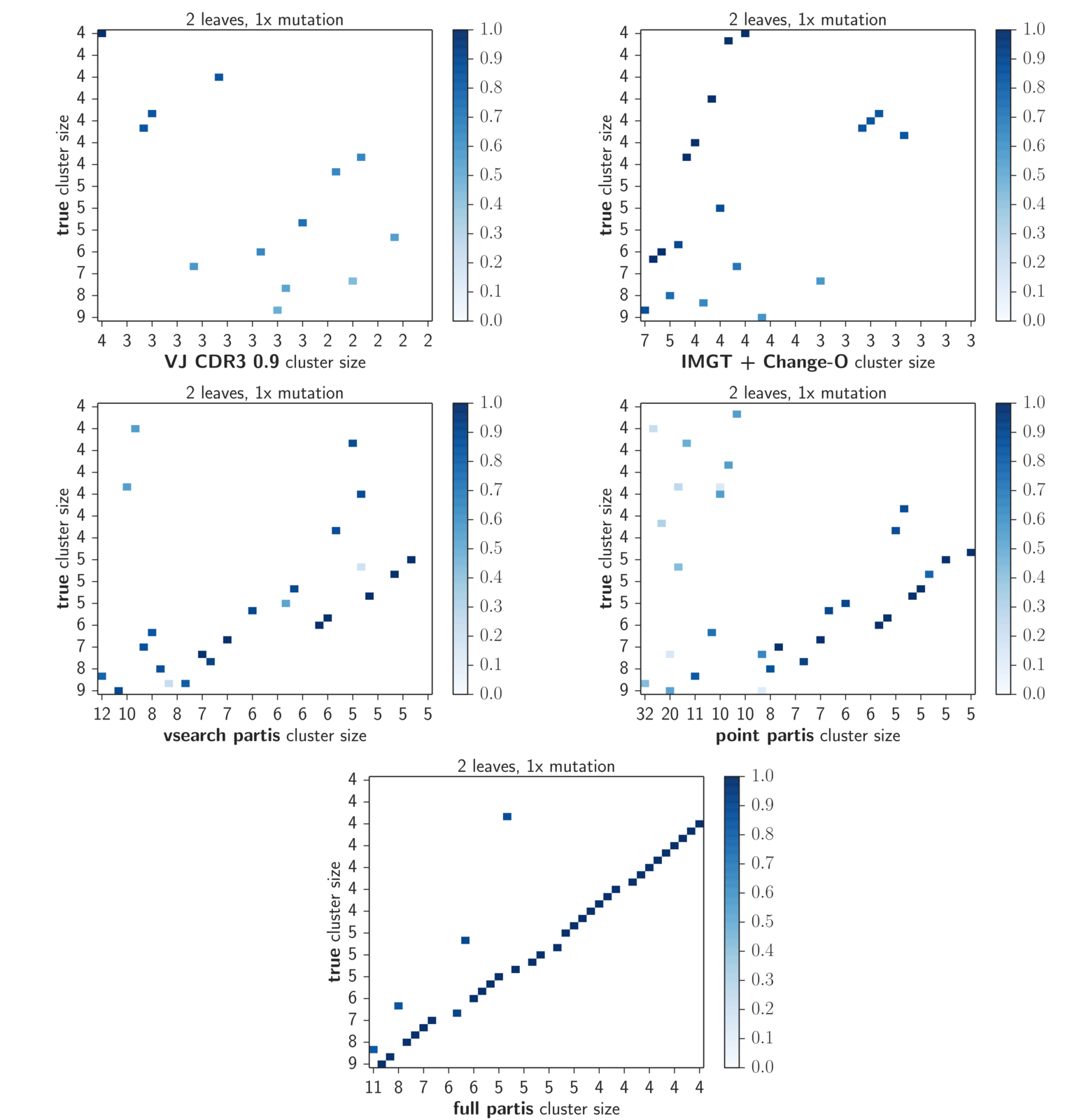

Supplement: S6 Fig — For these plots, we took the 40 largest clusters resulting from the given clustering and took their intersection with the 40 largest clusters generated by the simulation. Each non-white square indicates that there was a non-empty intersection between the two clusters; the square is shaded by the size of the clusters’ intersection divided by their mean size. The position of the square shows the relative sizes of the two clusters. (TIFF) [file pcbi.1005086.s006.tiff]

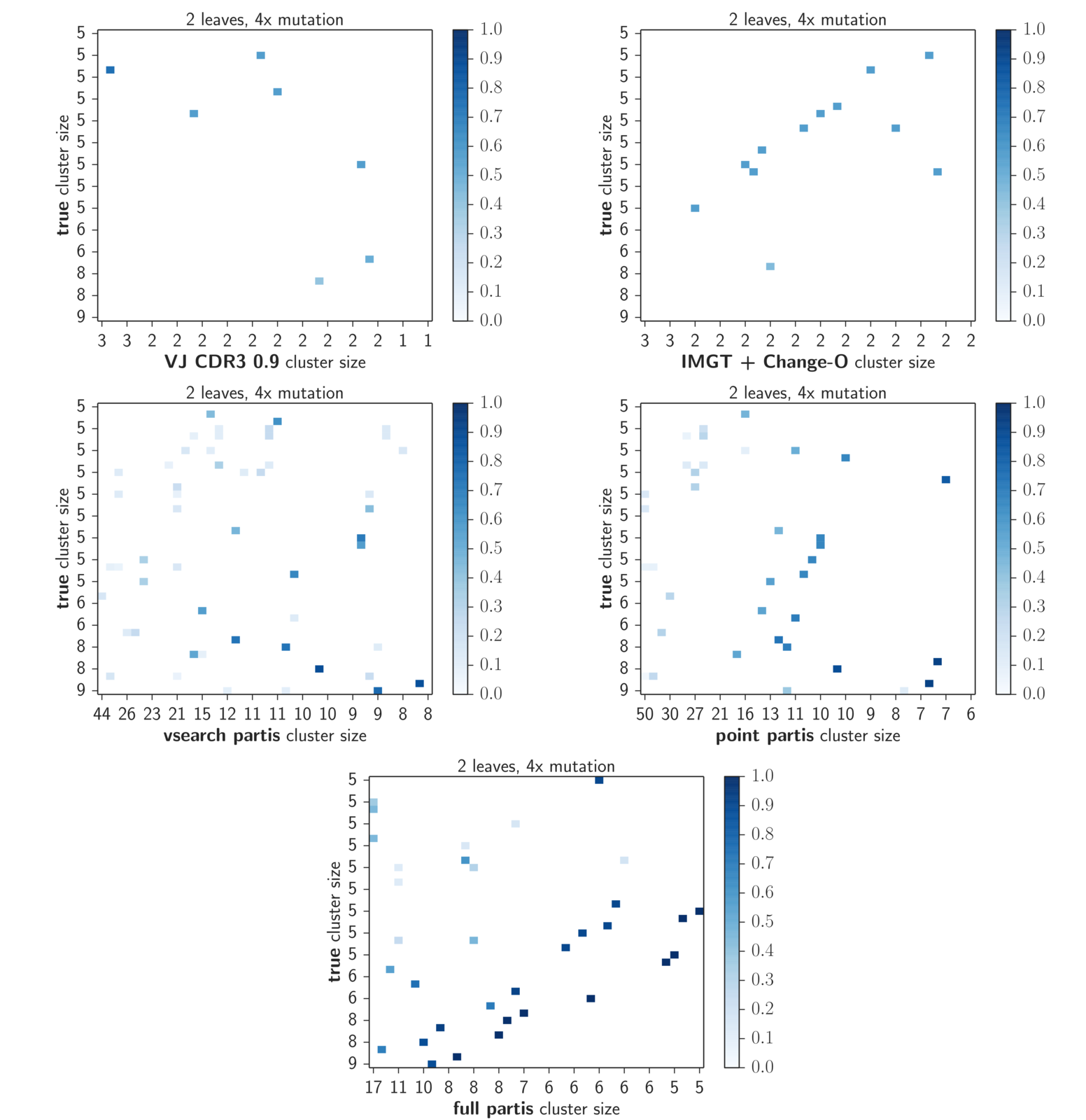

Supplement: S7 Fig — For these plots, we took the 40 largest clusters resulting from the given clustering and took their intersection with the 40 largest clusters generated by the simulation. Each non-white square indicates that there was a non-empty intersection between the two clusters; the square is shaded by the size of the clusters’ intersection divided by their mean size. The position of the square shows the relative sizes of the two clusters. (TIFF) [file pcbi.1005086.s007.tiff]

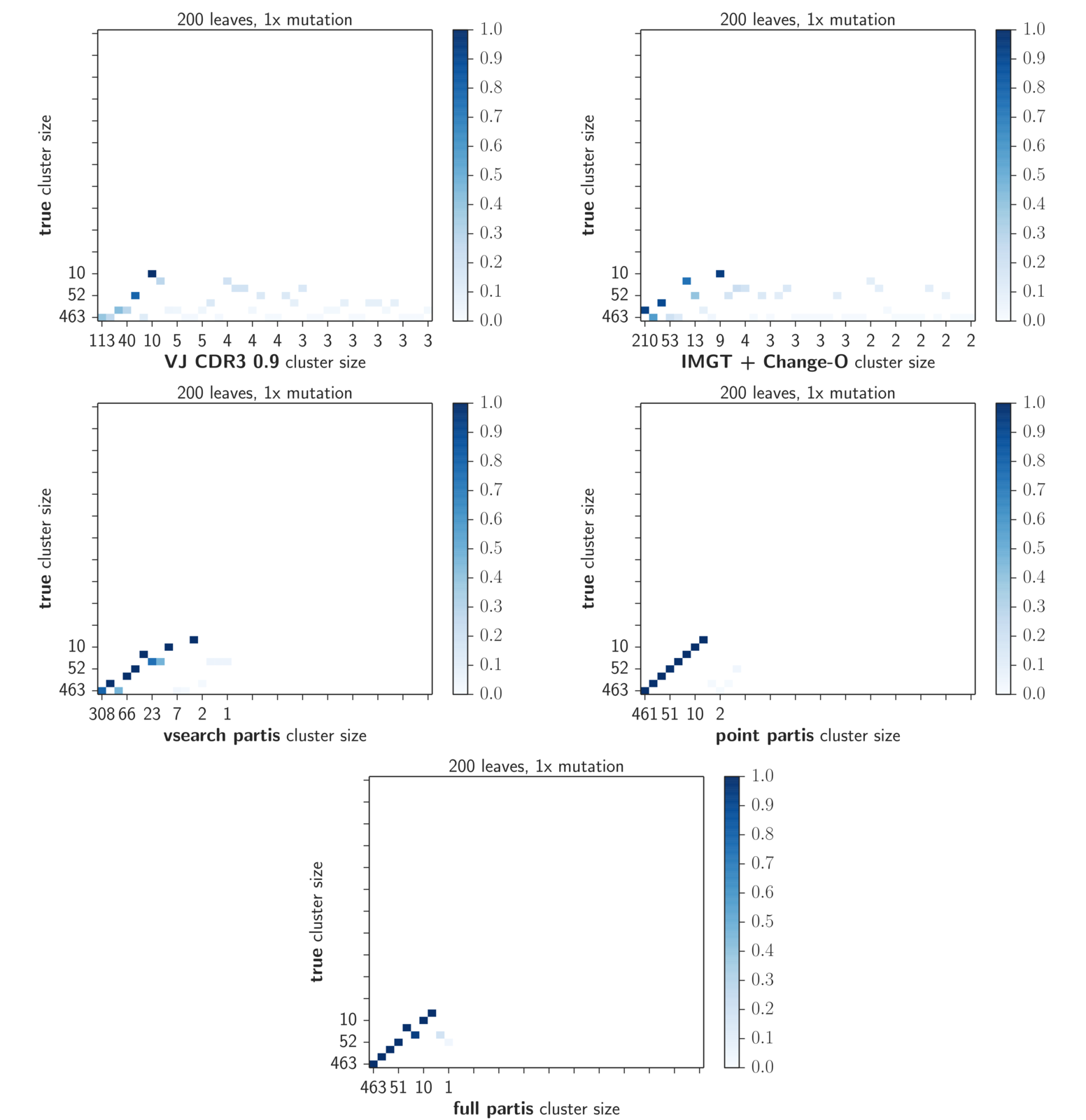

Supplement: S8 Fig — For these plots, we took the 40 largest clusters resulting from the given clustering and took their intersection with the 40 largest clusters generated by the simulation. Each non-white square indicates that there was a non-empty intersection between the two clusters; the square is shaded by the size of the clusters’ intersection divided by their mean size. The position of the square shows the relative sizes of the two clusters. (TIFF) [file pcbi.1005086.s008.tiff]

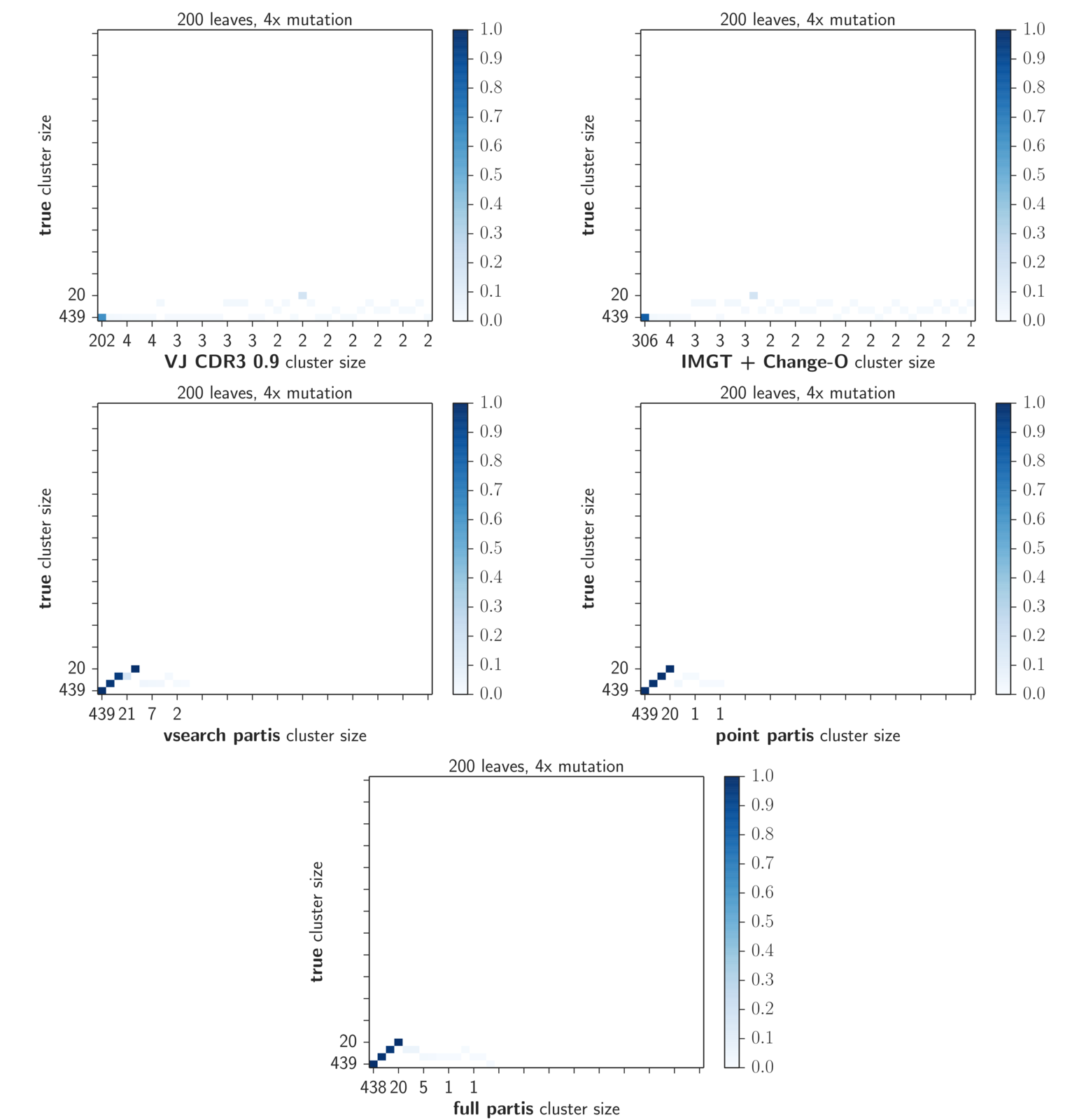

Supplement: S9 Fig — Results are shown for the simulation sample with a mean 200 geometric distribution for the number of leaves. For these plots, we took the 40 largest clusters resulting from the given clustering and took their intersection with the 40 largest clusters generated by the simulation. Each non-white square indicates that there was a non-empty intersection between the two clusters; the square is shaded by the size of the clusters’ intersection divided by their mean size. The position of the square shows the relative sizes of the two clusters. (TIFF) [file pcbi.1005086.s009.tiff]
